# Mother-to-child transmission of SARS-CoV-2 infection in high-income countries: a systematic review and meta-analysis of prospective observational studies

**DOI:** 10.1038/s41598-023-36097-1

**Published:** 2023-05-31

**Authors:** Daniela Morniroli, Giulia Vizzari, Martina Tosi, Giorgio Treglia, Antonio Corsello, Paola Marchisio, Fabio Mosca, Carlo Agostoni, Maria Lorella Giannì, Gregorio Paolo Milani, Lorenza Pugni

**Affiliations:** 1 Department of Clinical Sciences and Community Health, University of Milan, via della Commenda 12, 20122 Milan, Italy; 2 Fondazione IRCCS Ca’ Granda Ospedale Maggiore Policlinico, NICU, Milan, Italy; 3 Clinic of Nuclear Medicine, Imaging Institute of Southern Switzerland, Ente Ospedaliero Cantonale, 6500 Bellinzona, Switzerland; 4 Department of Nuclear Medicine and Molecular Imaging, Lausanne University Hospital, University of Lausanne, 1015 Lausanne, Switzerland; 5 Faculty of Biomedical Sciences, Università della Svizzera Italiana, 6900 Lugano, Switzerland; 6 Fondazione IRCCS Ca’ Granda Ospedale Maggiore Policlinico, S.C. Pediatria-Pneumoinfettivologia, Milan, Lombardia Italy; 7 Department of Pathophysiology and Transplantation, University of Milan, Milan, Italy; 8 Fondazione IRCCS Ca’ Granda Ospedale Maggiore Policlinico, Pediatric Unit, 20122 Milan, Italy

**Keywords:** Diseases, Health care, Risk factors

## Abstract

Mother-to-child transmission of SARS-CoV-2 has been reported since the onset of the COVID-19 pandemic. We conducted a study to summarize evidence on the risk of mother-to-child transmission in the first 30 days after birth in high-income countries and to evaluate the association between preventive measures and the risk of infection for the neonate. A systematic review and meta-analysis were undertaken following PRISMA guidelines. The National Library of Medicine, Web of Science, and Excerpta Medica databases were screened on February 26, 2022. All prospective observational studies addressing the frequency of infection in infants born to mothers SARS-CoV-2 positive were included. Twenty-six studies were included, reporting data of 2653 mothers with SARS-CoV-2 and 2677 neonates. The proportion meta-analysis pointed out an overall estimate of SARS-CoV-2 infection among infants of 2.3% (95% CI: 1.4–3.2%). Data from studies with (1.4%, 95% CI: 0.8–2) and without (1.3%, 95% CI: 0.0–2.7%) rooming-in provided similar risk of infection. Adopting at least two prevention measures during rooming-in resulted in a rate of mother-to-child infection of 1.0% (95%CI: 0.3–1.7%). The results of this study show a low rate of perinatal infection, support the rooming-in and confirm the effectiveness of preventive measures in reducing the risk of mother-to-child viral transmission.

## Introduction

Since its outbreak in March 2020, the COVID-19 pandemic has significantly impacted perinatal care. The main concern pointed out the possible transmission of the infection from a mother who tested positive for COVID-19 to her infant during the perinatal and early postnatal period^1,2^. This concern, justified by an initial lack of knowledge of the virulence and effects of this novel virus, led to the disruption of some practices recognized as crucial for maternal bonding and breastfeeding initiation, such as skin-to-skin and rooming-in^3^. This disruption in breastfeeding initiation, coupled with an initial concern about the viral transmission via breast milk or dyad proximity, has significantly impacted breastfeeding rates during the hospital stay and after discharge. As a result, increased reliance on breast milk substitutes occurred^4,5^. However, many authors have demonstrated the safety of breastfeeding even during maternal COVID-19 infection, and nowadays, these concerns and the choice of formulated milk seem unjustified ^6^. Furthermore, breastfeeding provides the optimum nutrition for the neonate and protects the infant against infection, even in the case of SARS-CoV-2^6,7^. Indeed, it has now been demonstrated that breast milk does not contain complete viral particles with the ability to replicate but instead contains antibodies with a neutralizing capacity in the case of a mother infected with the virus^8,9^. However, the duration and extent of this effect are still debated^10^. Due to the heterogeneity of studies to reduce the possibility of mother-to-child transmission, many scientific societies and hospitals have recommended that SARS-CoV-2 positive mothers adopt practices of distancing themselves from their babies and use personal protective equipment, such as masks and gloves during rooming-in or breastfeeding^11,12^. However, the effectiveness of these preventive measures in reducing mother-to-child transmission is still under study^11^. Within this complex context, gaining further insight into the rate of mother-to-child transmission of infection after birth, including the implementation of preventive measures such as wearing a mask and physical distancing in potentially decreasing the risk of infection, is critical to refine and adapting perinatal practices to the COVID-19 era. We conducted a systematic review to evaluate the infection transmission rate in the perinatal or early postnatal period in neonates born to mothers who tested positive for COVID-19 in high-income countries.

## Methods

To conduct this study, the 2020 edition of the Preferred Reporting Items for Systematic reviews and Meta-Analyses statement was followed^12^. The systematic review has been registered in the Research Registry (identifying number reviewregistry1640).

### Literature search strategy

The literature search was performed in the National Library of Medicine via PubMed, directly interfaced with MEDLINE, the National Library of Medicine’s database of citations from biomedical journals. We also performed literature search via Web of Science (WOS) and Excerpta Medica (Embase) databases on February 26, 2022.The following string was adopted: (newborns or neonates or infants) AND (SARS-CoV-2 OR COVID-19) AND (transmission OR infection OR horizontal) AND (mothers OR breastmilk OR breastfeeding OR human milk OR dyads OR rooming-in OR hygiene OR formula). The secondary references of all included articles were screened looking for additional reports. Three pairs of reviewers conducted the literature search in blind. At the end of the literature search, controversies were solved consulting a senior researcher.

### Eligibility criteria

Eligible were all prospective observational studies published in English addressing the frequency of infants born to mothers with a SARS-CoV-2 infection. The following inclusion criteria were considered: (1) SARS-CoV-2 infection in mothers had to be ascertained by a nasopharyngeal swab through a molecular test in the week preceding the delivery or on the day of delivery; (2) SARS-CoV-2 infection in infants had to be ascertained by a nasopharyngeal swab through a molecular test during the first 30 days after birth; (3) Studies had to be conducted in high-income countries as defined by World Bank classification. Studies including < 10 infected mothers were excluded. Date restriction was not applied.

### Data extraction and quality assessment

From each eligible study, the following information was extracted: first author’ name, year of publication, year of the subject’s enrolment, country, number of mothers with SARS-CoV-2 infection, number of infants with and without SARS-CoV-2 infection, clinical presentation (especially need of oxygen supplementation and/or ventilatory support) of infected mothers and infants, gestational age at delivery. Information on possible preventive measures included avoidance of rooming-in, distancing the neonate from the mother, breastfeeding avoidance, breastmilk pasteurization, the use of facial mask for the mother, hands hygiene and the use of gloves by mothers, respectively. The Newcastle–Ottawa Scale for assessing the quality of non-randomized studies in meta-analyses was used to assess the quality of the studies. Data extraction and quality assessment were independently performed by pairs of investigators. Controversies were solved by consensus. If controversies stood, a senior researcher was involved.

### Data synthesis and analysis

Data synthesis was performed through a proportion meta-analysis. The main outcome measure was the estimate of infected infants born to mothers with SARS-CoV-2 infection. Random-effects models were used. Pooled data were given with 95% confidence interval values (95% CI) and showed by forest plots. The I^2^-index was used to evaluate the heterogeneity and values > 50% were considered as significant. For the possible occurrence of a significant heterogeneity, subgroup meta-analyses focused on the use of rooming- in, on the adoption of at least two preventive measures and the year of subject’s enrollment were also planned. Publication bias was assessed through the use of Egger’s test. OpenMeta® software (Rockville, Maryland, USA) was used for the analyses.

A sensitivity analysis, excluding studies with extreme results, has also been performed to understand their influence on the main pooled analysis.

### Statement of ethics

An ethics statement is not applicable because this study is based exclusively on published literature.

## Results

### Literature search results

**Figure 1:**
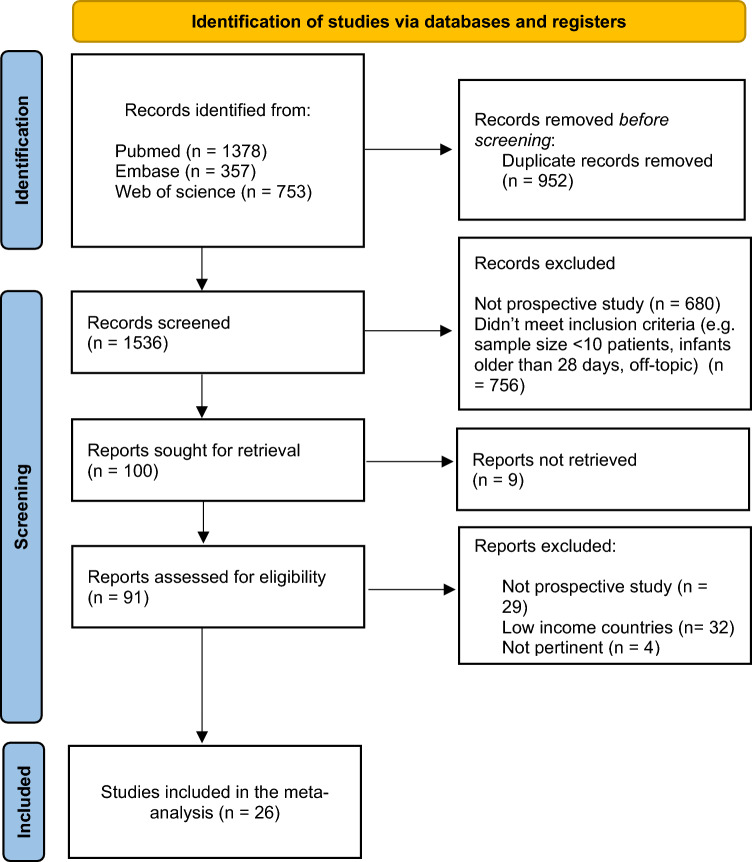
Flow diagram of the literature search process; *From* Page et al.^12^. For more information, visit: http://www.prisma-statement.org/.

The literature search process is shown in Fig. 1.

Among 1536 articles retained after duplication removal, we finally included 26 prospective studies^13–38^. Most studies (N = 21) were conducted during 2020, one in 2021 and four between 2020 and 2021 (Table 1).

**Table 1:** Study and patient characteristics.

Authors	Year of Enrolment	Country	Mothers with SARS-CoV-2 infection	Infants born to mothers with SARS-CoV-2	Infants with SARS-CoV-2	Rooming in	At least two preventive measures described
Szczygiol P et al	2021	Poland	83	84	7	Yes	No
Verma S et al	2020	USA	149	149	1	Yes	Yes
Zhang P et al	2020	USA	142	142	3	Not reported	No
Alouini S et al	2020–2021	France	45	46	1	Not reported	No
AlQurashi MA et al	2020	Saudi Arabia	45	45	0	No	Yes
Angelidou A et al	2020	USA	250	255	6	Yes	No
Biasucci G et al	2020	Italy	15	15	2	Yes	Yes
Blasco Santana L et al	2020–2021	Spain	29	32	1	No	No
Buonsenso D et al	2020	Italy	198	199	4	Yes	Yes
Villar J et al	2020	Multicenter	416	416	54	Not reported	No
Ibrahim CPH et al	2020	United Arab Emirates	71	72	2	Yes	Yes
Januszewski M et al	2020	Poland	47	48	0	Yes	No
Jimenez IM et al	2020	Spain	403	403	5	Yes	No
Kunjumon B et al	2020	USA	19	19	0	Yes	Yes
Ronchi A et al	2020	Italy	61	62	1	Yes	Yes
Rottenstreich A et al	2020	Israel	52	52	0	No	No
Salvatore CM et al	2020	USA	116	120	0	Yes	Yes
Shlomai NO et al	2020	Israel	53	55	0	No	Yes
Solís-García G et al	2020	Spain	73	75	1	Yes	Yes
Capozza M et al	2020	Italy	179	181	5	Yes	Not reported
Conti MG et al	2020–2021	Italy	33	33	0	No	Yes
Conti MG et al	2020–2021	Italy	28	28	3	No	Yes
Edlow AG et al	2020	USA	14	14	0	Not reported	Not reported
Falsaperla R et al	2020	Italy	64	64	1	Two groups (one yes and one no)	Two groups (one yes and one no)
Fenizia C et al	2020	Italy	30	30	2	Not reported	Not reported
Garcia-Ruiz I et al	2020	Spain	38	38	1	Not reported	Not reported

The studies were carried out in Italy (N = 8), in the USA (N = 6), in Spain (N = 3), in Poland (N = 2), in Israel (N = 2), in France (N = 1), in the United Arab Emirates (N = 1) and in Saudi Arabia (N = 1). One further study was an international multicenter study. The studies included 2653 mothers infected by SARS-CoV-2: only 4.7% (N = 126) of them needed oxygen or ventilatory support. A total of 2677 neonates were tested for SARS-CoV-2 and 100 resulted positive. Only five (5%) neonates needed oxygen supplementation and/or ventilatory support. All studies included infants born through vaginal and cesarean sections. In 14 studies the mother-infant dyad was enabled to stay together after birth 24 h a day^13,14,18,19,21,23–27,29,31,32,36^, in 6 it was not allowed^17,20,28,30,33,33^ and in the remaining 6 studies^15,16,22,35,37,38^, there was no information about it. In 12 studies two or more preventive measures were undertaken^14,17,19,21,23,26,27,29–31,33,34^, in 10 only one or no one^13,15,16,18,20,22,24,25,28,36^ and in the remaining 4 studies there was no information about preventive measures^32,35,37,38^. Considering only the studies in which rooming in practice was performed, at least two preventive measures were adopted in 8 of these studies^14,19,21,23,26,27,29,31^, one or no preventive measures were adopted in 5 studies^13,18,24,25,36^ and in one study it was not specified ^32^. The quality assessment evaluation is provided in the supplementary online table. We hereby provide a short summary of the NOS grading: Eight studies were considered as “high-quality” with a score ≥ 7, whereas twelve studies were considered “medium-quality”, having a score comprised between 4 and 6^15,18,20,24,25,34–36^.

### Proportion meta-analysis

**Figure 2:**
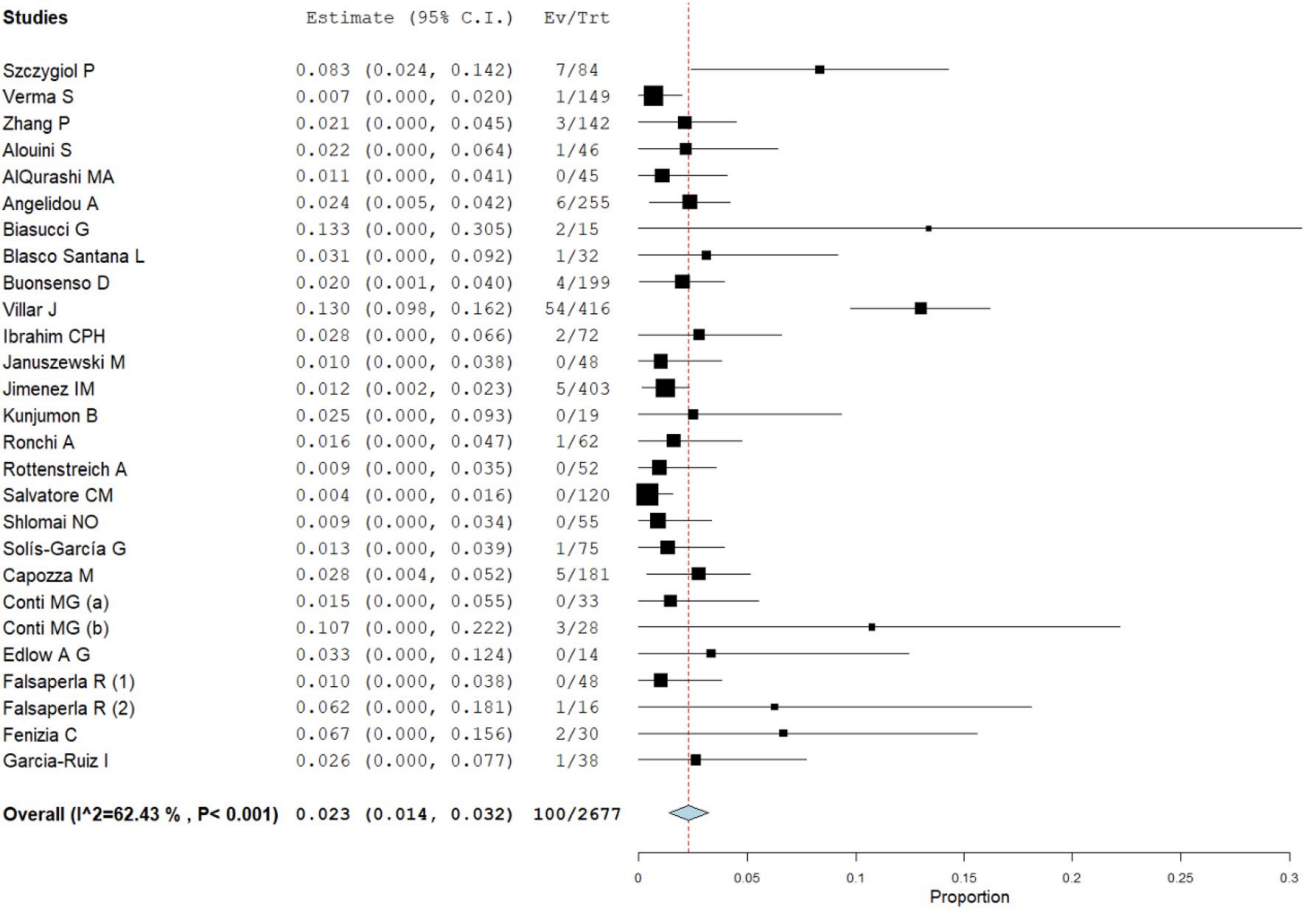
Proportion metanalysis of the overall estimate of SARS-CoV-2 infection among infants born to infected mothers.

**Figure 3:**
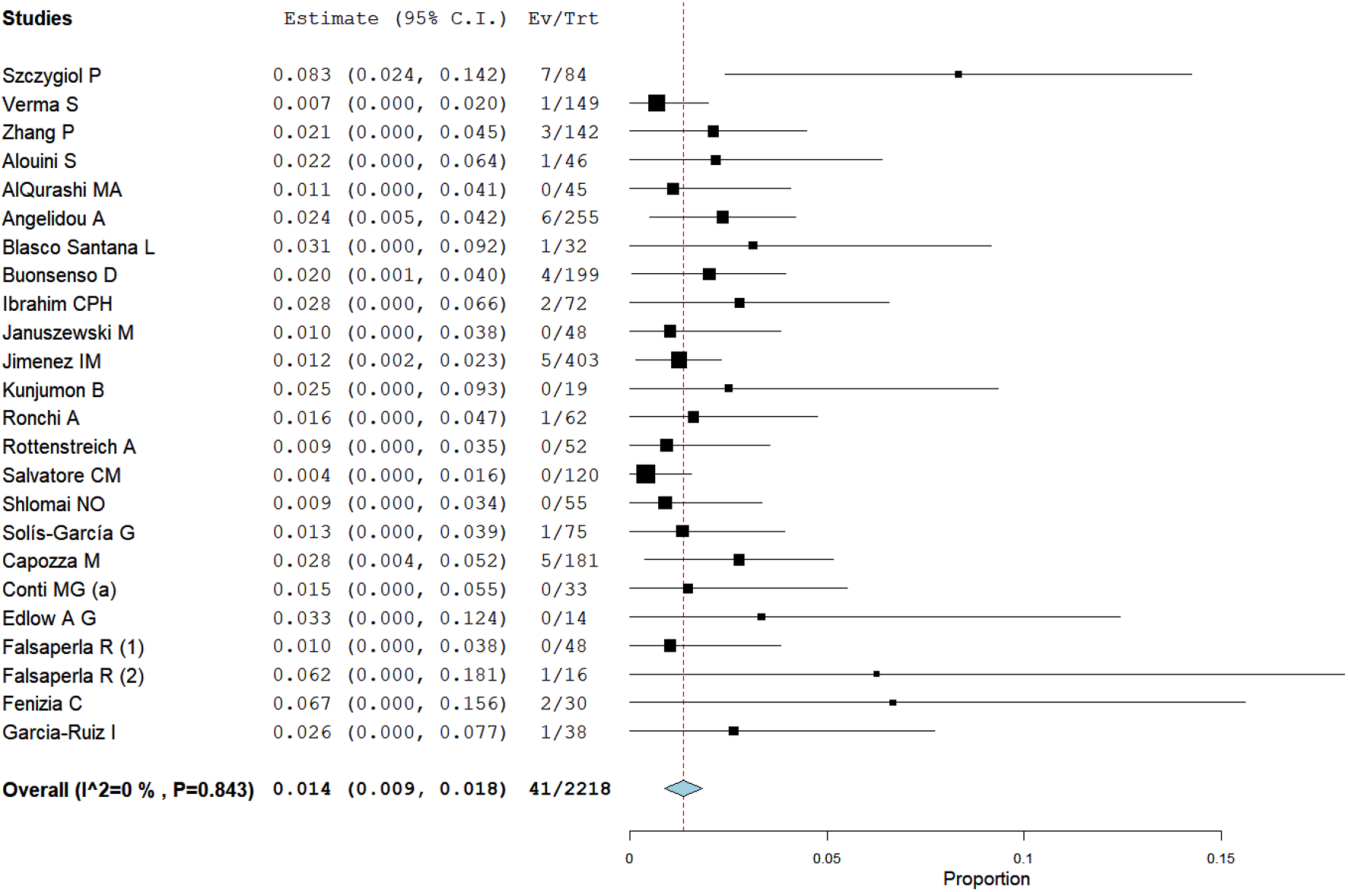
Proportion metanalysis of the overall estimate of SARS-CoV-2 infection among infants born to infected mothers (pooling data excluding the three studies with extreme results^19,22,34^).

The proportion meta-analysis pointed out an overall estimate of SARS-CoV-2 infection among infants born to infected mothers of 2.3% (95% CI: 1.4–3.2%), (Fig. 2). The I^2^-test was 62.4%, demonstrating a moderate heterogeneity. The sensitivity analysis performed excluding three studies with extreme results (pooled prevalence > 10%)^19,22,34^, showed a slight reduction of the overall pooled prevalence (1.4%, 95% CI: 0.9–1.8, I^2^-test 0%, Fig. 3) without a significant statistical difference compared to the main meta-analysis, taking into account the overlap of 95% confidence interval values among the two analyses.

**Figure 4:**
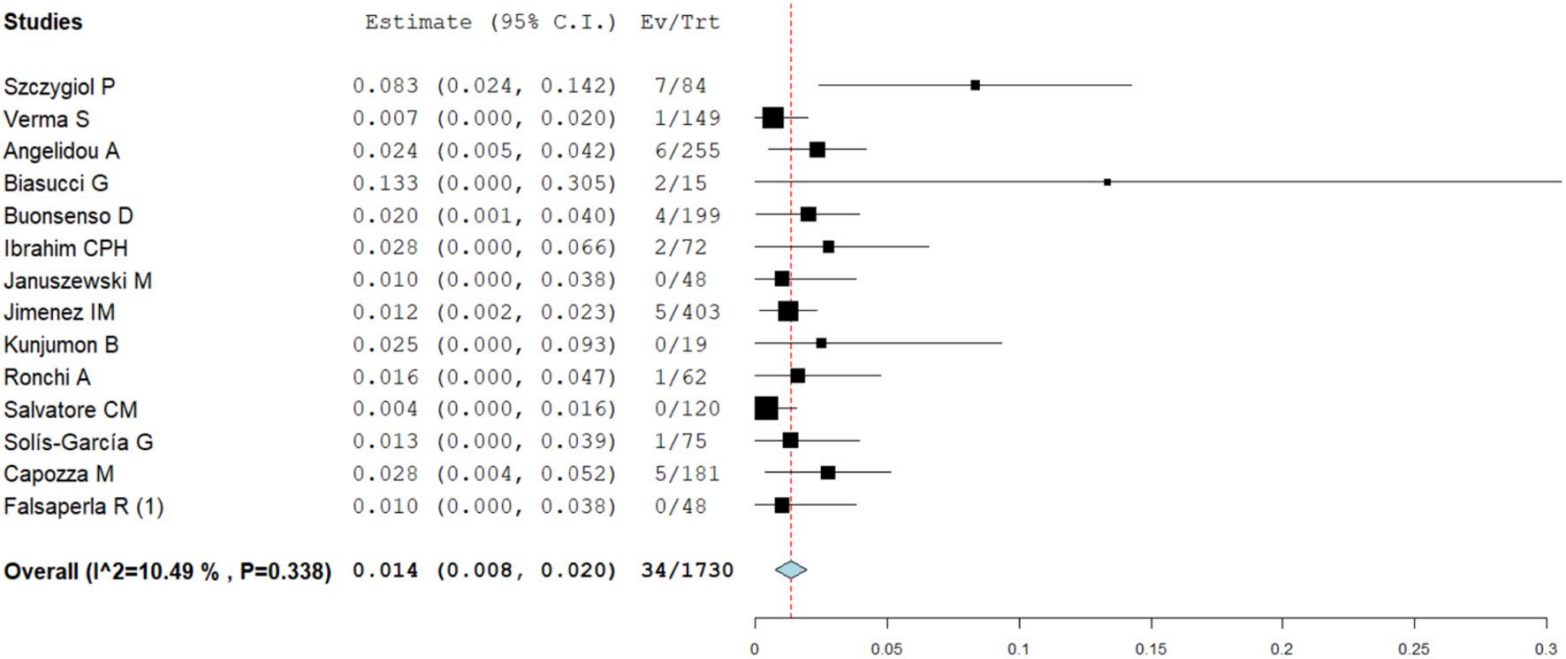
Sub-analysis investigating the relationship between rooming-in practice and the proportion of infected neonates (pooling data from studies with rooming-in).

**Figure 5:**
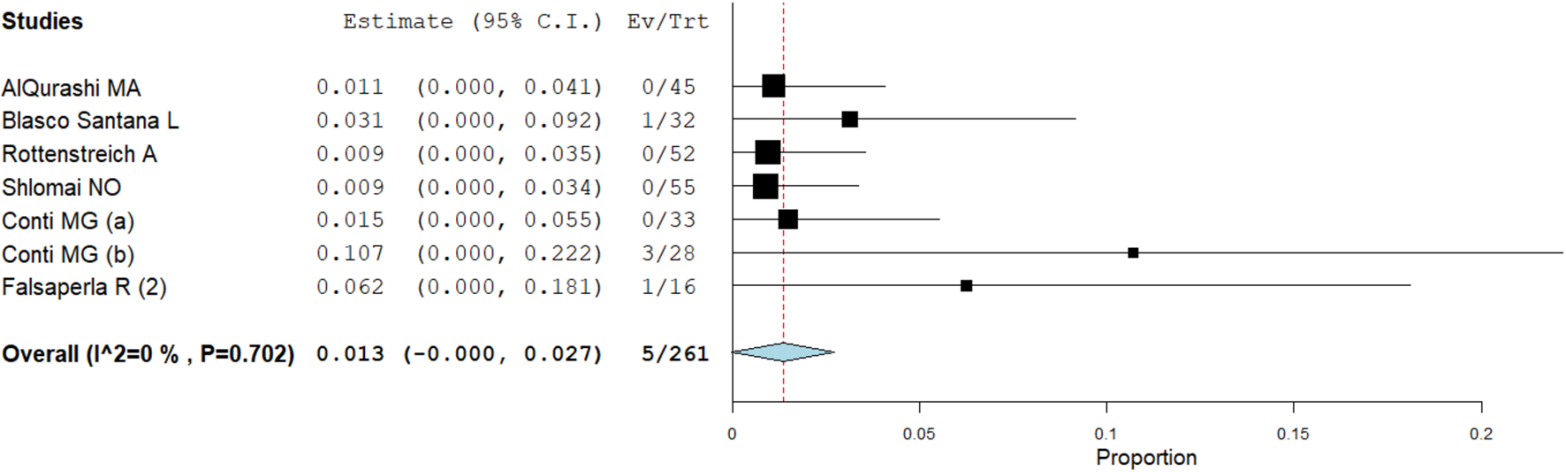
Sub-analysis investigating the relationship between rooming-in practice and proportion of infected neonates (pooling data from studies without rooming-in).

The sub-analysis investigating the relationship between rooming-in practice and the proportion of infected neonates showed similar data pooling from studies with (1.4%, 95% CI: 0.8–2%, I^2^-test 10.5%, Fig. 4) and without (1.3%, 95% CI: 0.0–2.7%, I^2^-test 0%, Fig. 5) rooming-in. The proportion meta-analysis pooling data from studies applying at least two preventive measures showed an infection estimate of 1.0% (95% CI: 0.4–1.7%, I^2^-test 0%), whereas data from studies with either one or no preventive measure provided an estimate of 3.2% (95% CI: 1.2–5.2%, I^2^-test 82%). Moreover, analyzing only data from studies with rooming-in, if at least two preventive measures were adopted, the infection rate was 1.0% (95%CI: 0.3–1.7%, I^2^-test 0%), while in the group with rooming-in and only one or no preventive measures the infection rate was 1.9% (95 CI: 0.8–3.0%, I^2^-test 31%). Finally, the sub-analysis including studies performed exclusively in 2020 showed similar results to that including studies performed also during 2021 (2.2%, 95% CI: 1.2–3.2%, I^2^-test 65.8%, vs 3.1 95% CI: 0.7–5.4, I^2^-test 35.8%, respectively). The latter data are shown in the supplementary online figures.

## Discussion

The objective of this systematic review and meta-analysis was to evaluate the proportion of infants born to mothers with COVID-19 who tested positive for SARS-CoV-2 during the first 30 days after birth. According to present findings, SARS-CoV-2 infection among neonates born to infected mothers was found to be 2.3%, indicating that mother-to-child transmission in the neonatal period appears to be relatively low and consistent with previously published data. The sensitivity analysis reported a mother-to-child transmission rate that, although slightly reduced, did not statistically differ from that found in the main meta-analysis, confirming the applicability of this study’s results. Moreover, the statistical heterogeneity was reduced (I^2^-test = 0%), confirming that, although the three studies with extreme results represented the source of the statistical heterogeneity in the main meta-analysis, they did not significantly influence the results of the pooled analysis.

The present meta-analysis collected data only from high-income countries with similar infant mortality and morbidity rates and a similar healthcare delivery system too. Despite the restriction in the inclusion criteria of the studies, the mother-to-child transmission rate of SARS-CoV-2 infection in the first month of life appears to overlap with that described in papers that included also low- and middle-income countries. Indeed, in the review by Gupta et al., the composite percentage of positive infants born to SARS-CoV-2 positive mothers appears to be between 1 and 4%^39^. Accordingly, Allotey et al. conducted a systematic review and meta-analysis including 472 studies (cohort studies, case series and case reports, respectively), independently of the prospective or retrospective data collection and countries’ income, and reported that the overall percentage of SARS-CoV-2 positivity in babies born to mothers with COVID- 19 was less than 2%^40^. Therefore our results indicate a similar infection rate when considering studies performed exclusively in 2020 vs studies performed exclusively in 2021, suggesting that the infection rate remains relatively low even in the presence of more transmissible variants of SARS-CoV-2, identified through 2021^41^. Indeed, since the first pandemic wave, there have been numerous variants of SARS-CoV-2, each with its own characteristics of virulence and contagiousness. In a further step, it will be worth understanding how the emergence of highly contagious variants (such as Omicron variants that appeared in 2021) may also have affected the percentage of infants who tested positive after birth. Among the neonates who tested positive for SARS-CoV-2 in the present study, only 5% needed oxygen supplementation and/or ventilatory support. This result is in line with several other studies which have reported a favourable outcome after delivery in most SARS-CoV-2 positive neonates^42–44^. As described in the systematic review by Garcia et al.^44^, symptoms of COVID-19 in the neonatal period appear to be mild, with a low mortality rate, mainly associated with the occurrence of comorbidities. Raschetti et al. reported that 55.7% of COVID-19 positive newborns were symptomatic, which is much higher than the percentage found by Garcia et el. and Liguoro et al., ranging between 11 and 20% ^42–44^. Differences in available studies could be due to the difficulty of discriminating whether a more severe clinical course is the result of an induced delivery or caesarean section imposed by the severe clinical condition of the mother, associated to neonatal complications connected to preterm delivery, mode of delivery and critical maternal conditions at the time of delivery, rather than to a real direct effect of the virus on the infants' organism^39,45^. In fact, our data support that the balance between the potential risk of postnatal transmission of the virus by the mother is decisively outweighed by the well-known benefits of skin-to-skin contact, rooming-in practice and above all, breastfeeding^6^. In fact, since the first wave of the pandemic, several international institutions, including the WHO, have issued guidelines that did not recommend the precautionary separation of the infected mother from her child^46,47^. These international policies had significant relevance in protecting the bonding of the dyad and the beneficial effects of breastfeeding even at a time when the lack of knowledge had led some government institutions in some individual countries to promote mother-baby separation ^48^. Given the rapidly changing recommendations on perinatal practices, particularly at the beginning of the pandemic and the incomplete consistency of perinatal policies with recommendations or best practices^11^, we conducted a sub-analysis to investigate whether the rooming-in practice was associated with an increased positivity rate of SARS-CoV-2 percentage in neonates born to mothers with COVID-19 infection. The results of the present study indicate that neonates who roomed in and those who were separated from their mother showed a similar rate of SARS-CoV-2 positivity, confirming the safety of the rooming-in practice whose major role in promoting maternal and infant bonding and breastfeeding is widely acknowledged^49^. On the contrary, Kollikonda et al.^50^ conducted a systematic review including 8 studies published before November 2020 and reported that 19.4% of roomed-in infants tested positive as compared to 1.67% of the isolated ones. The authors hypothesized that the high positivity rate associated with the practice of rooming-in could be partially due to inconsistent use of protective devices such as masks in addition to the implementation of inadequate air isolation measures. Moreover, considering that the practice of skin-to-skin contact with SARS-CoV-2 positive mothers was not associated with an increased positivity rate, a further speculation is related to the prolonged proximity to a SARS-CoV-2 positive mother that occurs during rooming-in rather than during the Kangaroo mother care. Indications of the WHO foresee that the mother should follow some preventive measures to reduce the probability of a transmitted infection. These measures mainly concern using face masks, gloves, recommendations on the distance to keep with the neonate, even if in the same room, and hand hygiene and washing^46^. The effectiveness of these measures in preventing an infection has been clearly demonstrated and has been widely recommended by almost all supranational institutions and local governments^51^. Concerning some particular situations, such as hospitalization after birth, the indications on which measures to adopt are less defined, and hospital policies often have determined their implementation in the first part of the pandemic^52^. Further, a sub-analysis showed that the use of two or more preventive measures correlated with a trend towards a reduction in the percentage of infants who tested positive during hospitalization (1% with two or more measures vs 3.2% with either one or no measures). Similarly, during rooming-in, adopting at least two preventive measures resulted in a rate of mother-to-child infection of 1% vs 1.9% if one or no preventive measures were taken. This observation further underlines the efficacy of implementing protective measures in terms of social and hospital life to limit the spread of the infection^51^. New variants of SARS-CoV-2 are still causing concern due to their very high contagiousness. At the same time, several governments have declared the end of the health emergency, removing some very effective preventive measures in the community, including the use of face masks and social distancing. While in maternity wards more and more women arrive at delivery positive for the virus, little is known about the effects of these highly contagious variants on neonates^39^. Our review presents at least some limitations. Firstly, nine studies could not be retrieved and therefore assessed for eligibility. We included even studies in which neonates were defined as infected or not considering the result of a single nasopharyngeal swab, although we were aware that, following the WHO indications, a single positive nasopharyngeal swab, especially in the early postnatal period, is not synonymous with certain infections^53^, and the timing of the neonate’s infection was not considered. Moreover, the results cannot be extended to low- and middle-income countries. Finally, it was not possible to accurately assess the risk of infection associated to the newer SARS-CoV-2 variants. On the other hand, we believe that there are also important strengths, since we included only prospective studies thus limiting the heterogeneity of the included reports. Moreover, we analysed the possible role of rooming-in and preventive hygiene measures in high-resource countries. In conclusion, the results of this meta-analysis confirm the low rate of SARS-CoV-2 perinatal infection in high-income countries. Moreover, based on the present findings, there is no reason to prevent infant-mother proximity, rooming-in and breastfeeding even in the event of a re-emerging new pandemic wave. In any case, preventive measures of viral transmission are crucial in containing the infection of the neonates looking forward towards a further reduction of adverse health outcomes in this peculiar fragile group, in the absence of data on the adverse effects of the infection on neurodevelopment and later stages of life.

## Supplementary Information

Supplementary Information.

## Data Availability

All data generated or analyzed during this study are included in this article and its supplementary material files. Further enquiries can be directed to the corresponding author.
