# Supplementary material for: Mother-to-child transmission of SARS-CoV-2 infection in high-income countries: a systematic review and meta-analysis of prospective observational studies
Source: Sci Rep. 2023 May 31;13:8813. doi: 10.1038/s41598-023-36097-1 (PMC10230471; doi:10.1038/s41598-023-36097-1)

Supplementary online material

Supplementary online Table. Newcastle-Ottawa Scale of each included study.

	Selection				*Comparability*		*Outcome*			
**Authors**	*Representativeness of exposed cohort*	*Selection of non-exposed cohort*	*Ascertainment of exposure*	*Demonstration that outcome of interest was not present at start of study*	*Adjust for the most important risk factors*	*Adjust for other risk factors*	*Assessment of outcome*	*Follow-up length*	*Loss to follow up rate*	***Total quality score***
Szczygiol P et al	*1*	*0*	*0*	*0*	*0*	*0*	*1*	*1*	*1*	***4***
Verma S et al	*1*	*0*	*1*	*0*	*1*	*0*	*1*	*0*	*0*	***4***
Zhang P et al	*1*	*0*	*1*	*1*	*1*	*1*	*1*	*1*	*1*	***8***
Alouini S et al	*1*	*0*	*0*	*0*	*1*	*0*	*0*	*0*	*0*	***2***
AlQurashi MA et al	*1*	*0*	*1*	*1*	*0*	*0*	*0*	*0*	*0*	***3***
Angelidou A et al	*1*	*1*	*1*	*1*	*1*	*1*	*1*	*1*	*1*	***9***
Biasucci G et al	*1*	*0*	*1*	*1*	*0*	*0*	*0*	*0*	*0*	***3***
Blasco Santana L et al	*1*	*1*	*1*	*0*	*1*	*1*	*1*	*1*	*0*	***7***
Buonsenso D et al	*1*	*0*	*1*	*0*	*0*	*0*	*1*	*1*	*1*	***5***
Villar J et al	*1*	*1*	*1*	*0*	*1*	*1*	*1*	*0*	*0*	***6***
Ibrahim CPH et al	*1*	*0*	*1*	*1*	*0*	*0*	*1*	*1*	*0*	***5***
Januszewski M et al	*1*	*1*	*1*	*1*	*1*	*1*	*1*	*0*	*0*	***7***
Jimenez IM et al	*1*	*1*	*1*	*1*	*1*	*1*	*1*	*0*	*0*	***7***
Kunjumon B et al	*1*	*0*	*0*	*1*	*0*	*0*	*1*	*0*	*0*	***3***
Ronchi A et al	*1*	*0*	*1*	*1*	*0*	*0*	*1*	*1*	*1*	***6***

Rottenstreich A et al	*1*	*0*	*1*	*1*	*0*	*0*	*1*	*0*	*0*	***4***
Salvatore CM et al	*1*	*0*	*1*	*1*	*0*	*0*	*1*	*1*	*0*	***5***
Shlomai NO et al	*1*	*0*	*1*	*0*	*0*	*0*	*1*	*0*	*1*	***4***
Solís-García G et al	*1*	*0*	*1*	*1*	*0*	*0*	*0*	*0*	*0*	***3***
Capozza M et al	*1*	*0*	*1*	*1*	*0*	*0*	*1*	*1*	*0*	***4***
Conti MG et al	*1*	*0*	*1*	*1*	*1*	*0*	*1*	*1*	*0*	***6***
Conti MG et al	*1*	*1*	*1*	*1*	*1*	*0*	*1*	*1*	*1*	***8***
Donati S et al	*1*	*0*	*0*	*0*	*0*	*0*	*1*	*0*	*0*	***2***
Edlow AG et al	*1*	*1*	*1*	*1*	*1*	*1*	*1*	*0*	*0*	***7***
Falsaperla R et al	*1*	*1*	*1*	*1*	*1*	*1*	*0*	*1*	*0*	***7***
Fenizia C et al	*1*	*0*	*1*	*1*	*0*	*0*	*0*	*0*	*0*	***3***
Garcia-Ruiz I et al	*1*	*0*	*1*	*1*	*0*	*0*	*0*	*0*	*1*	***4***

Supplementary online Figure 1. Proportion meta-analysis of estimate of infection among infants born to SARS-CoV-2 positive mothers in studies conducted in 2020.

**Studies**	Estimate (95% C.I.)	Ev/Trt
Verma S	0.007 (0.000, 0.020)	1/149
Zhang P	0.021 (0.000, 0.045)	3/142
AlQurashi MA	0.011 (0.000, 0.041)	0/45
Angelidou A	0.024 (0.005, 0.042)	6/255
Biasucci G	0.133 (0.000, 0.305)	2/15
Blasco Santana L	0.031 (0.000, 0.092)	1/32
Villar J	0.130 (0.098, 0.162)	54/416
Ibrahim CPH	0.028 (0.000, 0.066)	2/72
Januszewski M	0.010 (0.000, 0.038)	0/48
Jimenez IM	0.012 (0.002, 0.023)	5/403
Kunjumon B	0.025 (0.000, 0.093)	0/19
Ronchi A	0.016 (0.000, 0.047)	1/62
Rottenstreich A	0.009 (0.000, 0.035)	0/52
Salvatore CM	0.004 (0.000, 0.016)	0/120
Shlomai NO	0.009 (0.000, 0.034)	0/55
Solís-García G	0.013 (0.000, 0.039)	1/75
Capozza M	0.028 (0.004, 0.052)	5/181
Edlow A G	0.033 (0.000, 0.124)	0/14
Falsaperla R (1)	0.010 (0.000, 0.038)	0/48
Falsaperla R (2)	0.062 (0.000, 0.181)	1/16
Fenizia C	0.067 (0.000, 0.156)	2/30
Garcia-Ruiz I	0.026 (0.000, 0.077)	1/38
**Overall (I^2=65.84 % , P< 0.001)**	**0.022 (0.012, 0.032)**	**85/2287**

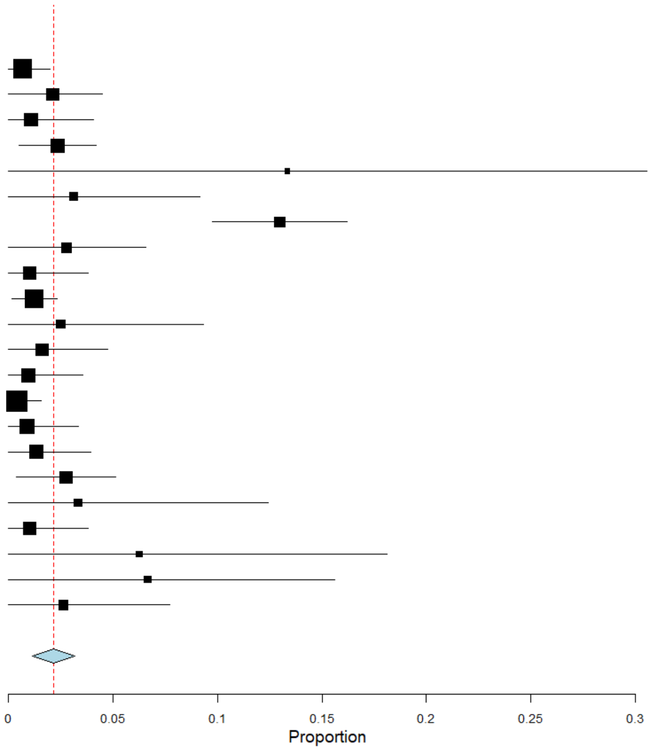

Supplementary online Figure 2. Proportion meta-analysis of estimate of infection among infants born to SARS-CoV-2 positive mothers in studies conducted up to 2021.

**Studies**	Estimate (95% C.I.)	Ev/Trt
Szczygiol P	0.083 (0.024, 0.142)	7/84
Alouini S	0.022 (0.000, 0.064)	1/46
Buonsenso D	0.020 (0.001, 0.040)	4/199
Conti MG (a)	0.015 (0.000, 0.055)	0/33
Conti MG (b)	0.107 (0.000, 0.222)	3/28
**Overall (I^2=35.77 % , P=0.183)**	**0.031 (0.007, 0.054)**	**15/390**

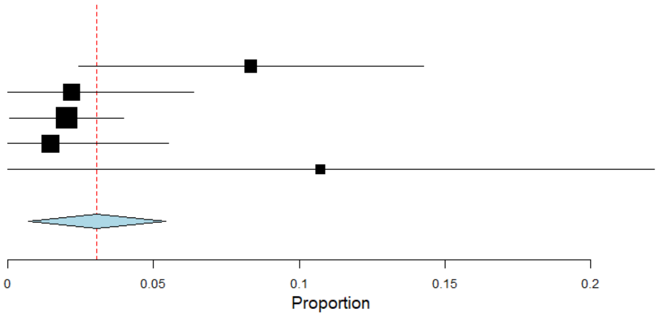

Supplement: Supplementary file 1 — Supplementary Information. [file 41598_2023_36097_MOESM1_ESM.pdf]
